# Dendritic Cell Vaccination in Non-Small Cell Lung Cancer: Remodeling the Tumor Immune Microenvironment

**DOI:** 10.3390/cells12192404

**Published:** 2023-10-04

**Authors:** Jensen Abascal, Michael S. Oh, Elvira L. Liclican, Steven M. Dubinett, Ramin Salehi-Rad, Bin Liu

**Affiliations:** 1 Division of Pulmonary and Critical Care, Department of Medicine, David Geffen School of Medicine at UCLA, Los Angeles, CA 90095-1690, USA; jensenabascal@ucla.edu (J.A.); michaeloh@mednet.ucla.edu (M.S.O.); eliclican@mednet.ucla.edu (E.L.L.); sdubinett@mednet.ucla.edu (S.M.D.); 2 Department of Medicine, VA Greater Los Angeles Healthcare System, Los Angeles, CA 90073, USA; 3 Department of Molecular and Medical Pharmacology, David Geffen School of Medicine at UCLA, Los Angeles, CA 90095-1690, USA; 4 Department of Pathology and Laboratory Medicine, David Geffen School of Medicine at UCLA, Los Angeles, CA 90095-1690, USA; 5 Jonsson Comprehensive Cancer Center, UCLA, Los Angeles, CA 90095-1690, USA

**Keywords:** non-small-cell lung cancer, NSCLC, tumor microenvironment, TME, dendritic cell, DC, DC vaccination, tumor vaccination, lung cancer, lung cancer vaccination, immunotherapy

## Abstract

Non-small-cell lung cancer (NSCLC) remains one of the leading causes of death worldwide. While NSCLCs possess antigens that can potentially elicit T cell responses, defective tumor antigen presentation and T cell activation hinder host anti-tumor immune responses. The NSCLC tumor microenvironment (TME) is composed of cellular and soluble mediators that can promote or combat tumor growth. The composition of the TME plays a critical role in promoting tumorigenesis and dictating anti-tumor immune responses to immunotherapy. Dendritic cells (DCs) are critical immune cells that activate anti-tumor T cell responses and sustain effector responses. DC vaccination is a promising cellular immunotherapy that has the potential to facilitate anti-tumor immune responses and transform the composition of the NSCLC TME via tumor antigen presentation and cell–cell communication. Here, we will review the features of the NSCLC TME with an emphasis on the immune cell phenotypes that directly interact with DCs. Additionally, we will summarize the major preclinical and clinical approaches for DC vaccine generation and examine how effective DC vaccination can transform the NSCLC TME toward a state of sustained anti-tumor immune signaling.

## 1. Introduction

Lung cancer is the leading cause of cancer-related death across the globe and the second most commonly diagnosed cancer [1]. Approximately 85% of lung cancers are histologically defined as NSCLC, which can be further classified as either squamous cell carcinomas (LUSC) or adenocarcinomas (LUAD) based on cell origin, morphology, and biological characteristics [2]. Tobacco smoke is the number one risk factor for NSCLC development [3]. Because of the mutagenicity of chemicals in tobacco smoke, NSCLCs have one of the highest mutational burdens of all malignancies [4]. These genomic mutations can potentially lead to the generation of cancer-specific mutant peptides capable of inducing tumor-specific T cell responses when appropriately presented in the context of major histocompatibility complex (MHC) molecules. DCs are professional antigen-presenting cells (APCs) that can activate both CD8^+^ and CD4^+^ T cells through the presentation of peptides on MHCI and MHCII molecules, respectively [5]. Typical antigen presentation of extracellular peptides is preceded by the phagocytosis or micropinocytosis of the antigen by the APC, antigen digestion into peptide fragments, peptide complex with the MHCII molecule, and the transportation of the MHCII–peptide complex onto the plasma membrane surface [6]. An alternative antigen presentation pathway known as cross-presentation enables peptides derived from the extracellular space to be complexed with the MHCI molecule. Cross-presentation is only necessary to initiate cellular immune responses when pathogens do not directly infect DCs. Antigen presentation via MHCII–peptide complexes enable DCs to prime CD4^+^ T cells to target extracellular pathogens such as bacteria and parasites, whereas DC cross-presentation via MHCI mediates the initiation of CD8^+^ T cell responses against intracellular threats such as viruses.

Defective antigen presentation in concert with the immunosuppressive milieu of the TME limit host anti-tumor immune responses. Cellular vaccination strategies utilizing DCs have been explored as a therapy to overcome immune suppression in the TME and promote immune-mediated tumor rejection. An increased density of DCs within the NSCLC TME is associated with improved clinical prognosis [7,8,9], and impaired DC function in the TME is associated with early stage NSCLC [10]. Because of the critical involvement of DCs in adaptive immune responses, their substantial crosstalk with immune cell populations, and their correlation with improved clinical outcomes, they are one of the most promising cellular vaccination strategies for NSCLC and other malignancies.

**Figure 1:**
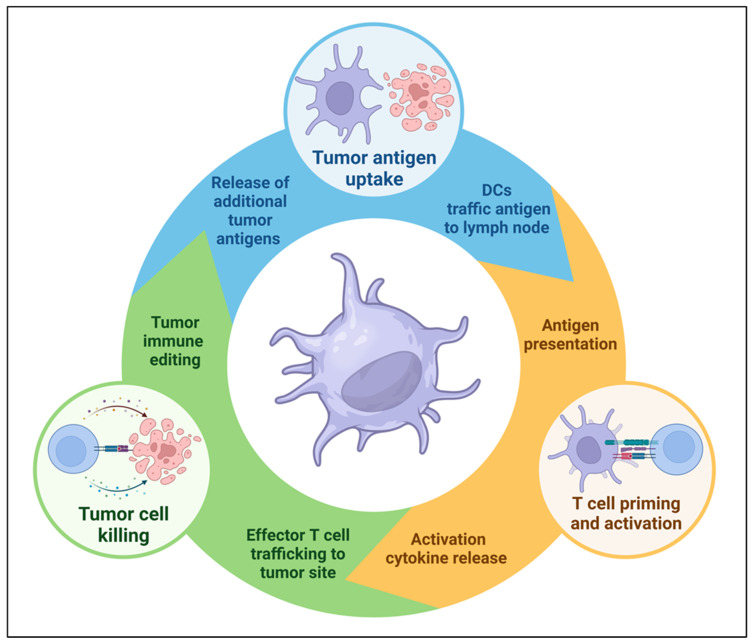
Dendritic cells play a key role in the cancer immunity cycle. DC vaccination can overcome deficiencies in the immunosuppressive tumor microenvironment (TME) that cause defective cycling and unrestrained tumor growth.

Modern technologies evaluating the spatial-temporal evolution of cell populations and signaling pathways in the TME have enhanced our understanding of the critical mediators of cancer immunity. The cancer immune cycle describes a seven-stage iterative process that is critical for the development of effective anti-tumor immune responses. These steps include: (1) release of cancer antigens, (2) cancer antigen presentation, (3) T cell priming and activation in tumor-draining lymph nodes, (4) effector T cell trafficking to tumors, (5) T cell infiltration into tumors, (6) the recognition of cancer cells, and (7) the killing of cancer cells [11]. DCs play a critical role at key stages of the cycle, and DC vaccination has the potential to augment multiple steps of the cancer-immunity cycle to facilitate host anti-tumor immune responses, including enhancing cancer antigen presentation, T cell priming and activation, and the release of additional cancer antigens from tumor cells targeted and killed by effector T cells (Figure 1).

This review focuses on the prominent cellular features of the NSCLC TME and summarizes how DC vaccination alters critical factors within the NSCLC TME. Additionally, an overview of the most commonly utilized DC vaccine culture techniques from both preclinical and clinical studies will be described. A deep understanding of the common TME signatures associated with effective DC vaccine responses can better inform future studies aiming to develop novel DC vaccination strategies for NSCLC.

## 2. DC Diversity and Function

### DC Subtypes and Functional Differences

Studies have demonstrated a high concordance between human and murine DCs, which can be divided into distinct subtypes on the basis of their development and function [12]. Human DCs originate from granulocyte–monocyte and DC progenitors (GMDP) that reside in the bone marrow (BM). The bifurcation of the DC developmental pathway is linked to the expression levels of interferon regulatory factor 8 (IRF8) in GMDPs [13]. IRF8^hi^ GMDPs develop into the common DC progenitor (CDP), which can give rise to three DC subsets: conventional DC 1 (cDC1), conventional DC 2 (cDC2), and plasmacytoid DCs (pDC). In contrast, IRF8^lo^ GMDPs develop into common monocyte progenitor (cMoP) cells that can further develop into monocytes. During inflammation, circulating monocytes upregulate DC machinery and can differentiate into monocyte-derived DCs (moDCs). An additional DC subtype known as DC3 has recently been identified, which has been shown to develop from IRF8^lo^ GMDPs [14].

cDC1s are the primary cross-presenting APCs, and they have been shown to be the principal DC subset responsible for tumor antigen trafficking and presentation to CD8^+^ T cells in vivo [15,16,17]. Within the murine cDC1 subtype, there are two varieties of cDC1: the lymph node resident CD8ɑ^+^ CD103^−^ cDC1 and the migratory CD8ɑ^−^ CD103^+^ cDC1 [18]. Recent studies have revealed that the optimal cDC1-mediated activation of CD8^+^ T cells depends on cDC1 “licensing” by activated CD4^+^ T cells via CD40/CD40L interactions [19]. cDC2s primarily function to present antigens to CD4^+^ T cells. cDC2 antigen presentation via MHCII to CD4^+^ T cells results in their activation and the secretion of inflammatory cytokines such as IFNγ [20], which can induce IL12 secretion from cDC1s and induce a feedback loop where CD4^+^, CD8^+^, and NK cells are further activated. This multi-cell network coordinates critical steps to initiate an optimal anti-tumor immune response signal and underscores the importance of a functional and diverse immune cell infiltrate within the TME.

pDCs secrete high amounts of type I IFNs such as IFNβ and are critical for immune responses against viruses [21]. pDC secretion of type I IFNs can enhance anti-tumor immune responses through macrophage activation, the stimulation of natural killer (NK) and CD8^+^ T cell cytotoxicity, and the promotion of DC maturation [22]. pDCs have also been shown to promote tumorigenesis; in one study, an elevated number of tumor-infiltrating pDCs secreting IL1ɑ was shown to promote tumor cell proliferation in NSCLC patients [23]. Due to their scarcity in vivo and the wealth of higher-value cell targets, pDCs remain an underexplored target of immunotherapy.

MoDCs are referred to as “inflammatory” DCs due to their accumulation and differentiation within inflamed tissues. While moDC differentiation driven by local inflammation has been observed in vivo, moDCs are also present within the tissues of healthy individuals not experiencing inflammation [24]. Like classical DCs, moDCs are capable of stimulating T cell proliferation and secreting cytokines such as TNFɑ, IL12p70, and IL23 [24]. moDCs differentiated ex vivo are the most commonly used DC subtype for human DC vaccination studies; moDC culture techniques and vaccination outcomes will be discussed in another section of this review.

Tumor-intrinsic mechanisms and a suppressive TME can hinder DC maturation to promote immune evasion through the development of tolerance [25]. Single-cell RNA sequencing (scRNA-seq) of human and murine NSCLC identified a new cluster of DCs—mature DCs enriched in immunoregulatory molecules (mregDCs) [26]—that express activation markers (CD40, CCR7, IL12β) and regulatory proteins (PD-L1, PD-L2, FAS), as well as proteins mediating a Th2 response (IL4Rɑ, IL4I1, CCL22). The mregDC phenotype is induced in both cDC1 and cDC2s after tumor antigen uptake and serves as a regulatory module susceptible to suppressive signals that limit DC functionality. The balance of pro- and anti-tumorigenic signals can change the fate of tumor antigen bearing DCs within the TME and distort their typical functions outside the context of cancer.

To appreciate the rationale for DC-based NSCLC vaccination, a fundamental understanding of the crosstalk between DCs and immune cells within the NSCLC TME is critical. DCs function at the interface between innate and adaptive immunity, often a bottleneck between an effective and ineffective anti-tumor immune response [27]. DCs engage in diverse cellular networks within the TME, and the signaling of these engagements can dictate the strength of an anti-tumor immune response. The following section highlights the major immune cell types within the NSCLC TME and their interactions with DCs.

## 3. Immune Cells in the NSCLC TME and Their Relationship with DCs

**Figure 2:**
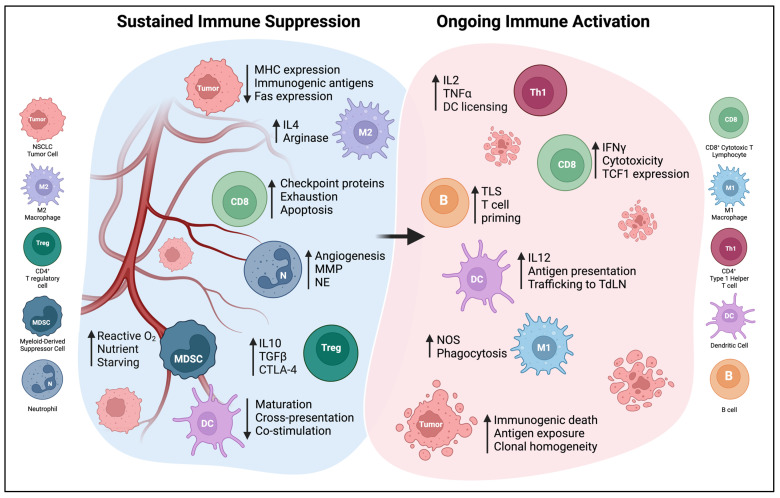
The NSCLC TME can be polarized by extreme immunosuppression or effective immune activation. Only some of these elements will be found in a given tumor, and the balance of these factors will determine the effectiveness of anti-tumor immunity. Abbreviations: MMP—matrix metalloproteinase, NE—neutrophil elastase, TLS—tertiary lymphoid structure, NOS—nitric oxide synthase, MHC—major histocompatibility complex, TdLN—tumor-draining lymph node.

The lung TME is a complex environment composed of cellular mediators such as macrophage, DC, NK, T, B, neutrophil, fibroblast, mesenchymal, and stromal cells, as well as soluble mediators and the extracellular matrix (Figure 2). DCs play an essential role in shaping the pro- or anti-tumorigenic signaling of the TME. Thus, it is critical to define the crosstalk between DCs and immune cells within the TME to understand the rationale for DCs as a therapeutic cancer vaccine. This section focuses on the heterogeneous and dynamic immune contexture of the NSCLC TME and highlights the diverging contribution of various immune subtypes to tumorigenesis and how DCs interact with these immune cells within the TME [28].

### 3.1. T Cells

The adaptive arm of the immune system can selectively kill tumors by identifying and targeting cancer cells expressing tumor-specific and/or tumor-associated proteins in a process known as immune surveillance. Cytotoxic CD8^+^ T cells are critical mediators of anti-tumor adaptive immunity [29]. After activation in tumor-draining lymph nodes, cytotoxic CD8^+^ T cells migrate to the TME to facilitate tumor killing. Cytotoxic CD8^+^ T cells kill cancer through two main mechanism: (1) the exocytosis of perforin and granzymes onto the plasma membranes of target cells and (2) the direct contact-mediated triggering of death receptors on cancer cells [30,31]. In NSCLC, increased CD8^+^ T cells within the tumor and surrounding stroma have been shown to be a favorable prognostic marker [32,33,34,35,36,37,38]. An increased presence of CD8^+^ CD103^+^ tissue-resident tumor-infiltrating lymphocytes (TILs), a highly activated subpopulation of effector T cells primed to carry out cytolytic effector functions, is associated with enhanced survival in patients with lung cancer [39]. Numerous NSCLC studies have concluded that a generalized increase in TILs is associated with improved survival and a decreased likelihood of systemic NSCLC recurrence [32,33,40].

Th1 CD4^+^ T cells are defined by their expression of the T-bet transcription factor and their ability to potently induce cytotoxic T lymphocyte (CTL) responses against intracellular threats such as viruses [41]. Th1 CD4^+^ T cells secrete cytokines such as IL2 and IFNγ, which promote effector T cell proliferation and induce MHC expression on the surface of cancer cells, respectively [42]. IL12 production by APCs is associated with increased Th1 responses and operates alongside IFNγ in a Th1-promoting feedback loop [43]. In a study of 170 NSCLC patients, increased Th1 polarization was associated with anti-tumor immune responses and better prognosis [44].

Th2 CD4^+^ T cells are defined by their expression of the GATA-3 transcription factor, and Th2 cytokine secretion is primarily composed of IL4, IL5, IL13, and IL10. It has been observed that Th2 cytokines such as IL10 can directly suppress DC-mediated antigen presentation and T cell activation; these observations have led to the longstanding hypothesis that CD4^+^ T cell Th2 polarization is a mechanism of tumor immune escape [45,46]. Recently, this hypothesis has been challenged by studies demonstrating that Th2 signaling promoting macrophage recruitment and a wound healing signature can induce potent anti-tumor immune responses [47,48,49].

Studies of the NSCLC TME have revealed that CD4^+^ regulatory T cells (Tregs) are strongly associated with poor survival [50,51,52,53,54]. Tregs are immunosuppressive and are typically identified through the expression of Forkhead box protein P3 (FOXP3), a transcription factor associated with Treg-specific gene expression [55]. Prostaglandin E2 (PGE2), an inflammatory factor produced in the Cyclooxygenase-2 (COX-2) pathway, has been shown to promote CD4^+^ Treg FOXP3 expression and regulatory functions [56,57]. There are several mechanisms of Treg-mediated immune suppression, including the secretion of inhibitory cytokines such as IL10 and transforming growth factor beta (TGFβ), the expression of immunosuppressive surface proteins such as Cytotoxic T-lymphocyte associated protein 4 (CTLA-4), and the overconsumption of IL2 within the TME [58]. In a NSCLC preclinical study, the depletion of Tregs via an antibody targeting CD25, a surface protein found on a majority of CD4^+^ Tregs, synergized with carboplatin chemotherapy and significantly extended the survival of tumor-bearing mice compared to chemotherapy alone [59].

Persistent exposure to tumor antigens can lead to T cell exhaustion. Originally observed in patients with chronic viral infections [60], T cell exhaustion is the gradual epigenetic reprograming of T cells, which results in progressive loss of function [61]. T cell exhaustion results in an increased co-expression of inhibitory receptors such as Programmed death protein 1 (PD-1), Lymphocyte-activation gene 3 (LAG3), T cell immunoglobulin and mucin-domain containing-3 (Tim-3), T cell immunoreceptor with Ig and ITIM domains (TIGIT), and CTLA-4. An increased co-expression of inhibitory receptor proteins is associated with the transition from early T cell exhaustion (defined by minimal functional defects) to terminal exhaustion and the complete loss of T cell functionality [61]. A high expression of the thymocyte selection-associated HMG BOX (TOX) transcription factor is associated with elevated inhibitory receptor expression and T cell exhaustion [62]. Elevated TOX expression in tumor-infiltrating T cells is highly predictive of reduced overall survival in both NSCLC and melanoma [63].

Immune checkpoint blockade (ICB) therapies that disrupt the binding between exhaustion proteins and their ligands have yielded significant clinical benefits, shifting the treatment paradigm for many cancer types, including NSCLC [64,65,66]. Therapeutics disrupting the PD-1/PD-L1 axis have been approved to treat several different cancers [67]. A landmark study identified a population of Tcf1^+^ PD1^+^ CD8^+^ T cells as an undifferentiated population of intratumoral T cells critical for anti-tumor immune responses following anti-PD-1 ICB [68]. This study, along with others carried out in NSCLC and melanoma preclinical models [69,70], support the hypothesis that anti-PD-1 ICB acts to reinvigorate non-terminally exhausted T cell subsets to control tumor growth. Currently, a new generation of inhibitory molecules that target diverse checkpoint pathways and aim to expand the ICB treatment options for patients with NSCLC and other malignancies are under development [71].

Effective T cell and DC interactions are critical for an optimal anti-tumor immune response. Naïve T cell activation initiated by receptor–ligand binding between the T cell receptor (TCR) and peptide–MHC complex on the surface of DCs is the critical first step that enables a self-renewing T cell response against cancer or pathogens [72]. DCs and other APCs are the only immune cells capable of providing naïve T cells with a co-stimulatory signal. The binding of CD28 on the surface of the naïve T cell with CD80 or CD86 on the surface of an activated DC provides a key secondary signal that promotes T cell survival, cytokine production, and proliferation. In the context of an anti-tumor immune response, the absence of DCs capable of providing productive TCR stimulation or co-stimulation can lead to a TME devoid of tumor-specific T cells and unchecked tumor growth [73].

The importance of DC and T cell interactions extends beyond the initiation of T cell activation. Intratumoral CD103^+^ cDC1s produce high amounts of CXCL9/10 chemokines upon type I IFN autocrine signaling [74,75]. CXCL9/10 chemokine gradients attract effector T cells expressing the CXCR3 chemokine receptor [76]. CXCL10 production by CD103^+^ cDC1s was shown to be required for endogenous and adoptively transferred effector T cells migration into tumor sites, suggesting that the absence of intratumoral cDC1s may contribute to tumor immune escape mechanisms and limited responses to ICB [77]. Furthermore, in a meta-analysis of over one thousand cancer patients with seven different types of cancer, CXCL9 expression was one of the strongest predictors to ICB response [78]. The efficacy of ICB therapy targeting PD-L1 has been shown to be dependent on PD-L1^+^ DCs. In a murine model of colorectal carcinoma, DC-conditional PD-L1 knockout abrogated anti-PD-L1 efficacy, and this effect was phenocopied in *Batf3*^−/−^ mice lacking cDC1s [79]. These results suggest that PD-L1^+^ DCs may be the primary immune cells engaging PD-1^+^ T cells and limiting T cell functionality. A better understanding of the stimulatory and inhibitory interactions between DCs and T cells is necessary to fully appreciate the potential for DCs to regulate T cell function within the TME.

### 3.2. B Cells and Tertiary Lymphoid Structures

The role of B cells within tertiary lymphoid structures (TLS) and the prognostic value of TLS within NSCLC tumors has been a flourishing field of focus within the biology of lung cancer. Mature TLS contain T cell- and B cell-dense follicles enriched with high endothelial venules that facilitate their migration [80]. TLS do not form in healthy tissue and are often identified in the context of chronic inflammation, including cancer [81]. Numerous studies have demonstrated that the presence of TLS within or adjacent to NSCLC is positively prognostic [7,82,83]. Both B cells and antibody-producing plasma cells can be found within TLS, and studies of patients with NSCLC have shown a significant correlation between intratumoral plasma cell density and overall survival after anti-PD-L1 ICB [84]. Due to their significant functional and organizational overlap with lymph nodes, anti-tumor immune responses can arise from T cell priming within TLS rather than traditional priming within tumor-draining lymph nodes (TdLNs) [85]. This priming is driven by DCs within TLS presenting tumor antigens to T cells, and a combined measurement of follicular B cells and LAMP^+^ mature DCs within TLS was the best predictor of survival for both early- and late-stage NSCLC [8]. In LUSC, a high density of TLS is a positive prognostic marker associated with increased patient survival [86]. Interestingly, the positive prognosis of intratumoral TLS was lost after neoadjuvant chemotherapy, which was attributed to defects in B cell germinal center (GC) formation within the TLS due to corticosteroid treatment within the chemotherapy regime. This finding emphasizes the importance of B cells within TLS and suggests that TLS lacking GC B cells do not exert the same anti-tumorigenic immune control as mature TLS containing GC B cells. These data highlight the need for more studies to better understand anti-tumor immune responses driven by TLS within NSCLC.

pDCs have been shown to regulate the expression of Toll-like receptor 7 (TLR7) on the surface of B cells through the secretion of type 1 IFN [87]. The activation of TLR7 promotes B cell expansion, and pDC secretion of IL6 further induces B cell differentiation into antibody-producing plasma cells [88]. DCs have been shown to augment B cell antibody secretion. CD40^+^ DCs activated by CD40L significantly enhance IgG and IgA secretion by B cells, and IgM secretion is significantly increased in the presence of both IL2 and DCs [89]. DC-mediated enhancement of B cell antibody secretion has been observed in NSCLC tumors containing TLS. In a study of twenty-seven patients with NSCLC tumors containing TLS, 44% displayed antibody reactivity against at least one tested tumor antigen, a finding that supports the link between DC-positive TLS and tumor-reactive antibodies [8].

### 3.3. Macrophages

Macrophages can be polarized as either M1 or M2, which is directly connected to arginine metabolism [90]. M1 macrophages produce nitric oxide synthase (NOS), which converts arginine to nitric oxide, while arginase produced by M2 macrophages metabolizes arginine into ornithine and urea. M1 macrophages drive CD4^+^ Th1 polarization and are associated with promoting cytotoxic T cell responses, and M2 macrophages polarize CD4^+^ T cells toward a Th2 humoral response [91]. However, recent scRNA sequencing studies have shown significant overlap between M1 and M2 gene signatures and alternative states of macrophage polarization [92]. These studies propose that macrophage polarization is best described as a spectrum of functionality and rely on lineage tracing to define macrophage subtypes independent of their functional state [93].

Increased numbers of macrophages within tumor islets correlate with an improved survival benefit in patients with NSCLC [35,94,95], and an increase in intratumoral M1 macrophages is associated with the best overall survival [96]. Although some studies have not shown a correlation between outcomes and M2 tumor infiltration in patients with NSCLC, others have demonstrated that M2 macrophage infiltration can significantly shorten patient survival [96,97,98,99]. M2 macrophage infiltration within LUAD is correlated with increased intratumoral microvessel density, which is a poor prognostic indicator, suggesting that increased M2 macrophage abundance contributes to angiogenesis in NSCLC [99,100]. In addition, M2 macrophage abundance within NSCLC tumors has been shown to increase NSCLC metastasis by inducing an epithelial to mesenchymal transition (EMT) and promoting tumor cell proliferation [101,102].

The lung microenvironment harbors a tissue-specific macrophage subtype known as lung tissue-resident alveolar macrophages (AMs), which serve to preserve host defense, clear pulmonary surfactants, and maintain overall lung homeostasis [103]. AMs reside alongside NSCLC throughout its progression, and the polarization and function of AMs can dictate the fate of a lesion. Multiple studies have shown impaired AM cytotoxicity within NSCLC tumors and unaltered circulating macrophage cytotoxicity simultaneously, suggesting that components of the NSCLC TME can dampen AM cytotoxicity and enable tumor progression [104,105]. AMs have been shown to promote a pro-tumorigenic NSCLC microenvironment via direct the secretion of anti-inflammatory IL10 or the enhancement of TGFβ secretion by tumor cells [106,107]. In addition, NSCLC tumorigenesis has been shown to preferentially arise in close proximity to AMs, and NSCLC EMT can be accelerated by neighboring AM signaling [108]. Due to their unique presence within the TME of NSCLC and capacity to promote tumor progression, further studies aiming to improve our understanding of AMs role throughout tumorigenesis are needed to better understand NSCLC biology and inform treatment options.

The macrophage-mediated suppression of DC function has been documented within the TME of NSCLC and other malignancies. IL10 secretion by macrophages has been shown to downregulate the production of IL12 by DCs within the TME and blunt CD8^+^ T cell-mediated anti-tumor immune responses [109]. IL10 can also directly inhibit TLR signaling within DCs, resulting in severely attenuated APC functionality [110]. The macrophage-dependent inhibition of DC activation has also been observed within healthy lungs. Studies of LPS-induced asthma have demonstrated that macrophage-derived IL10 dampens DC inflammatory responses in the majority of individuals who do not develop an allergic response to inhaled LPS [111]. AMs have been shown to reduce the number and functionality of DCs within tumor-bearing mice, resulting in the downregulation of MHCII and CD80/86 on the surface of DCs within the lung [112]. Furthermore, an ex vivo study of AM inhibitory functions demonstrated that the addition of AMs significantly reduced the DC-dependent induction of cell proliferation in mixed lymphocyte reactions (MLRs) [113]. Interestingly, the inhibition of the nitric oxide synthase pathway in AMs eliminated their suppressive activity, a result that was independently verified in a similar study [114].

### 3.4. Neutrophils and Myeloid-Derived Suppressor Cells

Neutrophils are the most common circulating leukocyte in blood. The composition and function of tumor-associated neutrophils (TANs) throughout NSCLC tumor progression is a growing field of study. Though early studies of TANs revealed two major neutrophil subsets categorized as either pro-inflammatory N1 or anti-inflammatory N2 [115], this binary classification of N1/N2 neutrophil polarization has since been revised to reflect transcriptional substates within pro- and anti-tumor neutrophils. Additionally, a neutrophil subtype is included within the broader category of myeloid-derived suppressor cells (MDSCs), known as polymorphonuclear MDSCs (PMN-MDSCs, or granulocytic-MDSCs, or G-MDSCs). PMN-MDSCs are identified as CD11b^+^ Ly6G^+^ Ly6C^lo^ in mice and as CD11b^+^ CD14^−^ CD15^+^ in humans [116,117]. scRNA sequencing analysis of TANs in murine and human lung cancers revealed five neutrophil subsets in human and six in murine tumors [12]. Despite this difference in neutrophil subset numbers in humans and mice, TAN phenotypes are conserved between species.

Pro-tumor TANs can promote tumor progression through several mechanisms, including T cell suppression, the promotion of angiogenesis, and the secretion of enzymes that accelerate tumor cell proliferation [118]. In a meta-analysis of nearly four thousand cancer patients from twenty different studies, elevated neutrophil content within the TME was shown to be independently associated with poor overall survival [119]. In addition, an elevated neutrophil to lymphocyte ratio (NLR) is associated with poor NSCLC prognosis and has been utilized to stratify ICB responsiveness [120,121]. Soluble factors in the TME, such as TGFβ and Granulocyte colony-stimulating factor (G-CSF), have been shown to program TANs to suppress anti-tumor T cell responses [122,123,124]. Arginase-1 secretion by TANs limits T cell proliferation and constrains T cell survival and anti-tumor effector function [125]. Matrix metalloproteinase (MMP) secretion by TANs promotes NSCLC angiogenesis and enables cancer cells to migrate to sites of metastasis [126,127]. TANs within NSCLC can promote NSCLC proliferation through the secretion of PGE2 and neutrophil elastase (NE), a serine protease capable of destroying bacterial and host tissues at the site of inflammation [128]. In a murine model of NSCLC, the CXCR2-mediated recruitment of neutrophils and subsequent secretion of NE was shown to promote tumor growth and angiogenesis [129].

Despite the wealth of publications illustrating the immunosuppressive nature of TANs, anti-tumor TANs can inhibit tumor growth through the secretion of cytokines capable of inducing T cell expansion and activating NK cells and DCs [115,130]. Preclinical studies in the LKR-13 adenocarcinoma model identified TGFβ as a suppressor of anti-tumor TAN function, and an antibody blockade of TGFβ resulted in a significant expansion of anti-tumor TANs indispensable for subsequent anti-tumor immune responses [122]. A separate preclinical NSCLC study determined that anti-CD40 immunotherapy response was dependent on a subset of anti-tumor TANs with an interferon gene signature and elevated CXCL10 secretion [131]. Similarly, a preclinical metastatic breast cancer study found that IFNγ enhanced TAN tumoricidal function and prevented breast cancer metastasis development within the lungs [132]. A study in murine and human melanoma demonstrated that neutrophils are responsible for complete responses to ICB and destroy tumor cells that survive T cell killing via antigen escape [133].

The expression of the lipid metabolism-related molecule lectin-type oxidized LDL receptor-1 (Lox-1) distinguishes PMN-MDSCs from neutrophils in patients with cancer [134]. A NSCLC study aiming to identify the circulating biomarkers associated with anti-PD-1 response found that an elevated NK cell/Lox-1^+^ PMN-MDSC ratio predicted clinical response to anti-PD-1 [135]. This elevated ratio was also correlated with improved overall survival and progression-free survival in patients with NSCLC. Similarly, a preclinical study found that the depletion or functional inhibition of PMN-MDSCs within murine liver kinase B1^−/−^ (*Lkb1*^−/−^) NSCLC models can re-sensitize tumors to anti-PD-1 ICB therapy [136].

A separate subset of MDSCs known as monocytic MDSCs (M-MDSCs) are derived from monocytes [116,117]. M-MDSCs are identified as CD11b^+^ Ly6G^−^ Ly6C^hi^ cells in mice and CD11b^+^ CD14^+^ HLA-DR^−/lo^ CD15^−^ cells in humans [116]. The mechanisms of immune suppression carried out by M-MDSCs share significant overlap with pro-tumor TANs and PMN-MDSCs. M-MDSCs can deprive T cells of nutrients via L-arginine and L-cysteine consumption, leading to the inhibition of T cell proliferation and reduced effector functions [137]. M-MDSC signaling can also influence T cell polarization. For example, TGFβ and retinoic acid produced by human MDSCs can skew T cell polarization to FoxP3^+^ Tregs [138]. MDSCs can also directly inhibit T cell functions via an increase in the production of reactive oxygen and nitrogen species, which can disrupt TCR and IL2R signaling, resulting in inert T cells [139,140]. A NSCLC clinical study found that a subset of tumor-infiltrating B7-H3^+^ CD14^+^ HLA-DR^−/low^ M-MDSCs secrete elevated amounts of IL10 and are associated with reduced recurrence-free survival [141]. M-MDSCs not only promote tumor progression but also limit anti-tumor immune responses in a variety of treatment settings. CD14^+^ HLA-DR^−/low^ M-MDSCs were shown to be associated with progressive disease and poor responses to platinum-doublet chemotherapy in patients with NSCLC [142]. MDSC immune suppression is not specific to NSCLC; in a meta-analysis of sixteen studies with approximately two thousand patients across various types of cancer, increased MDSC frequency was found to be associated with decreased overall survival and disease-free survival [143].

Similar to their dualistic role of being either pro- or anti-tumorigenic, neutrophils are capable of potently activating or suppressing DCs in the TME. Neutrophils have been shown to selectively cluster with immature DCs via interactions between the neutrophil surface protein Mac-1 (CD11b/CD18; alpha M beta 2) and the DC surface protein DC-SIGN [144]. Once in proximity, TNFɑ secretion by activated neutrophils can stimulate DC maturation and promote downstream T cell activation. Ex vivo co-culture experiments have revealed that the presence of neutrophils promotes CD86, CD40, and MHCII upregulation on the surface of DCs, and Mac-1 mediated cell–cell contact between neutrophils and DCs is required for CD86 and MHCII upregulation [145]. Neutrophils have also been shown to indirectly promote DC activation. Neutrophils activated by TLR ligands promote NK cell locomotion to sites of inflammation through the secretion of IL8, and the clustering of neutrophils and NK cells results in NK cell activation and the upregulation of the NK activation marker CD69 [146]. NK cells not exposed to neutrophils exerted minimal effect on DCs in co-culture experiments, but DCs co-cultured with NK cells previously activated by neutrophils upregulated CD86, CD83, and MHCII and simultaneously secreted significant amounts of IFNγ, TNFɑ, and IL12.

While the examples above demonstrate the capacity of neutrophils to activate DCs within the TME, several studies have shown that PMN-MDSCs and M-MDSCs can suppress DC antigen presentation. Previous studies have demonstrated that the accumulation of oxidized lipids can limit DC cross-presentation, and one recent study revealed that PMN-MDSCs are capable of suppressing DC cross-presentation through the direct transfer of oxidatively truncated lipids [147]. The pharmacological inhibition of the myeloperoxidase pathway in PMN-MDSCs restored DC cross-presentation, suggesting that drugs targeting the myeloperoxidase pathway have the potential to restore DC cross-presentation and enhance anti-tumor immune responses. Tumor-derived MDSCs have also been shown to inhibit DC MHCII antigen presentation. A mix of M- and PMN-MDSCs derived from either a melanoma or pancreatic murine cancer model inhibited DC antigen presentation to CD4^+^ T cells through a nitric oxide (NO)-dependent mechanism [148]. This study identified that the signal transducer and activator of transcription 1 (STAT1) protein, which must be phosphorylated at Tyrosine 701 during DC antigen presentation, is instead nitrated at Tyrosine 701 in patients with melanoma or pancreatic cancer. Previous studies by the same group implicated NO as a mediator of STAT1 nitration, which suggests that MDSC-derived NO could be nitrating STAT1 in DCs to inhibit antigen presentation within the TME [149,150].

## 4. DCs as Cancer Vaccines

DCs are indispensable for the initiation and maintenance of an adaptive anti-tumor immune response. The rationale for NSCLC DC vaccination is two-fold: DCs themselves can effectively present tumor antigens to prime T cell-mediated immune responses, and DCs’ engagement with other immune cells within the TME bolsters anti-tumor immune signaling. The vaccination of NSCLC and other malignancies with functional DCs has been an intense field of study for decades. Since the first human DC cancer vaccination study published in 1995, the technology of DC vaccination has advanced considerably [151]. Our improved understanding of DC immunobiology and the establishment of new culture techniques has led to promising clinical responses and advanced methods to improve DC functions prior to injection. Here, we will provide an overview of the most commonly utilized methods to generate DCs for NSCLC and other tumor vaccination therapies.

### 4.1. Murine BMDCs

DC vaccination studies treating murine models of NSCLC have provided key evidence that the therapy is safe and viable, in addition to revealing the dominant mechanisms that dictate improved anti-tumor immune responses (Table 1). This evidence has informed clinical trial design and shaped the methods utilized to gather data supporting DC vaccination efficacy. While DC vaccine therapy has shown encouraging results in the clinic, there have been challenges translating the robust responses seen in murine models to human disease.

Early preclinical vaccination studies utilized bone marrow (BM)-derived DCs (BMDC) [170], which are generated by incubating BM precursors in the presence of Granulocyte-macrophage colony-stimulating factor (GMCSF) and IL4 [171]. BMDCs resemble mouse cDC2s found in vivo, and both depend on the expression of the IRF4 transcription factor for proper development [172]. The vaccination of murine models of NSCLC with BMDCs has led to tumor regression and the induction of tumor-specific T cell responses [152,153,154]. BMDCs are amendable to gene modification through gene-silencing siRNA or infection with viral vectors encoding tumor-associated antigens (TAAs) or immunomodulatory proteins [155,157,158,159,160,161,162,163,164,173]. These modifications have been shown to improve immune responses compared to control DC treatment. Despite these promising findings, a study of murine BMDC cultures found that they are composed of both macrophages and dendritic cells, a result that has rendered this heterogeneous culture less desirable for DC vaccination research [172].

### 4.2. MoDCs

The overwhelming majority of DC vaccine clinical trials treating NSCLC and other cancers have utilized MoDC culture protocols [174]. Similar to BMDCs in mice, human MoDCs are differentiated from patient PBMCs cultured in the presence of GMCSF and IL4. MoDC culture can also be initiated from a more homogenous monocyte population using CD14^+^ cells isolated from PBMCs [175]. Unlike murine BMDCs, human MoDC cultures result in a homogenous cell population with an elevated expression of the DC-SIGN surface marker [176]. A cytokine cocktail composed of TNFɑ, IL1β, IL6, and PGE2 has been shown to promote MoDC maturation and the upregulation of canonical APC activation markers such as CD86 and CD83 [177]. In addition, functional assays have shown that MoDCs can uptake and efficiently cross-present cell-associated antigens to activate T cells [178,179]. Considering the evidence supporting cross-presentation as a critical step in initiating cytotoxic anti-tumor immune responses, this feature makes MoDCs a suitable candidate for human DC vaccine studies.

Despite the prevalence of MoDC vaccination and numerous studies demonstrating their ability to induce tumor-specific immune responses, their efficacy in the clinic has been limited [180]. Studies have investigated the potential of genetic manipulation to augment MoDC effectiveness as a clinical therapeutic. Prior to their intratumoral administration as part of a phase 1 clinical trial, MoDCs genetically modified with an adenovirus-expressing CCL21 were thoroughly characterized [181]. CCL21 (also known as secondary lymphoid tissue chemokine, SLC) functions to attract naïve T cells and antigen-experienced DCs to lymphoid tissues by binding to CCR7 on the cell surface [182]. This characterization revealed that the MoDCs could survive viral transduction and secrete elevated concentrations of CCL21. Additionally, the transduction did not disrupt the key biological functions of the MoDCs, including cytokine secretion, phagocytosis, and antigen presentation. mRNA electroporation is an alternative method that has been used to genetically modify moDCs. Studies have shown that the activation of NF-κβ via the electroporation of constitutively active Iκβ kinases promotes moDC IL12p70 secretion and NK cell activation [183,184]. Additional studies profiling the vaccine efficacy of genetically modified MoDCs have been conducted [185,186], and as genetic manipulation techniques advance, substantial modifications of the DC genome could become standard practice in the DC vaccine field.

### 4.3. Stem Cell-Derived DCs

Induced pluripotent stem cell-derived DCs (iPSC-DCs) and embryonic stem cell-derived DCs (ES-DCs) are murine DC culture approaches that allow for the generation of DCs that closely resemble cDC2s [187]. This preclinical culture technique involves plating stem cells onto the murine OP9 BM feeder cell line for the first five days of culture, and the subsequent addition of GMCSF differentiates the stem cells into DCs [188]. iPSC-DC intratumoral vaccination has been shown to synergize with radiotherapy (RT) to generate robust anti-tumor immunity in AT-3 and B16 preclinical cancer models bearing poor immunogenicity [165]. This culture technique remains highly experimental, and future studies profiling the efficacy and anti-tumor immune responses associated with iPSC-DC/ES-DC vaccination in NSCLC are needed.

### 4.4. cDC1s

Two main methodologies have been employed to culture murine cDC1s from BM precursors. The first, termed iCD103-DCs, utilizes a culture protocol similar to BMDCs with the addition of recombinant FMS-like tyrosine kinase 3 ligand (FLT3L) cytokine [189]. Murine BM cells cultured in the presence of FLT3L and GMCSF for fifteen days differentiate into iCD103-DCs through the activation of BATF3, ID2, and IRF8, the same transcription factors that dictate murine cDC1 cell development in vivo. The second protocol utilizes modified OP9 feeder cell lines that express the Notch ligand Delta-like 1 (OP9-DL1), a ligand involved in cDC1 development in vivo [190]. Murine BM cells are co-cultured with the OP9-DL1 feeder cell line, along with physiological concentrations of FLT3L for seven days, which drives the differentiation of BM precursors into CD103^+^ cDC1s with a high degree of phenotypic and functional similarity to their in vivo counterparts [191]. However, this protocol typically leads to lower yields than the iDC103-DC culture method.

The generation of a robust human cDC1 culture technique is a field of intense research, while a clinical trial utilizing ex vivo-generated human cDC1s as a vaccine has not yet been conducted, major steps toward a clinical grade human cDC1 culture have been made [192]. Recently, a clinically applicable culture technique that generates a heterogeneous mixture of pDC, cDC1, and cDC2s from human CD34^+^ progenitor cells was published [193]. The cDC1s generated from this culture exhibited substantial overlap with natural cDC1 cells found in circulation and were capable of antigen cross-presentation. Notably, this culture technique is not limited by cDC1 yield; approximately four million cDC1s can be generated from a starting culture of one million blood-derived CD34^+^ progenitors. Further research into optimizing the functions and yields from human cDC1 cultures will lead to landmark clinical trials treating malignancies with cDC1s generated ex vivo.

### 4.5. Naturally Circulating DCs

Preclinical studies have isolated conventional DC subtypes from murine blood and lymphoid tissue for tumor vaccination [194]. Because DCs are rare within the blood, mice are often pre-treated with FLT3L to expand endogenous DCs prior to collection. A pre-clinical melanoma DC vaccine study expanded endogenous DCs by subcutaneously injecting a B16 melanoma tumor engineered to secrete FLT3L (B16-FLT3L), and expanded splenic CD8^+^ cDC1s were collected and used as a vaccine. These cDC1s cross-presented the OVA antigen to endogenous CD8^+^ T cells in vivo and potentiated anti-PD-1 efficacy in both MC38 and B16/F10 preclinical tumor models [166]. In 2007, a study investigated whether a combination vaccine composed of two natural DC subtypes—pDCs and myeloid DCs (mDCs)—could synergize to enhance anti-tumor effects against the preclinical E.G7.OVA murine lymphoma cell line. Donor mice pre-treated with plasmid DNA encoding the FLT3L protein exhibited a near ten-fold increase in pDC and mDCs, and the pDC + mDC combination exhibited superior anti-tumor efficacy compared to either DC subset alone [169]. Clinical trials utilizing natural DC vaccines composed of cDC2s or pDCs, or even a combination of the two, have shown tumor-specific immune responses in patients with stage III melanoma, a result that highlights the capacity for DC vaccination to induce immunological responses at an advanced disease stage [195,196].

### 4.6. Translation to the Clinic

The only FDA-approved DC vaccine for human cancer is a natural DC vaccine known as Sipuleucel-T, which is used to treat castration-resistant prostate cancer [197]. DCs are harvested from patients via leukapheresis and incubated with a fusion protein of prostatic acid phosphatase (PAP) prostate antigen and GMCSF, which matures the DCs and helps direct immune responses against prostate cancer cells expressing high amounts of PAP in vivo. The Sipuleucel-T vaccine is composed of a heterogenous mixture of cells, but the hypothesized mechanism of action is thought to depend on productive PAP antigen presentation by DCs within the vaccine product to endogenous T cells within patients. This natural DC vaccine has been shown to significantly increase patient survival by a median of 4.1 months, and patients treated with Sipuleucel-T have approximately a 23% reduced risk of death compared to placebo treatments [197]. The success and approval of Sipuleucel-T is a major achievement in the field of DC cancer vaccination and illustrates the clinical viability of this modality of cancer therapy.

## 5. Microenvironmental and Systemic Changes Induced by DC Vaccine Therapy

**Figure 3:**
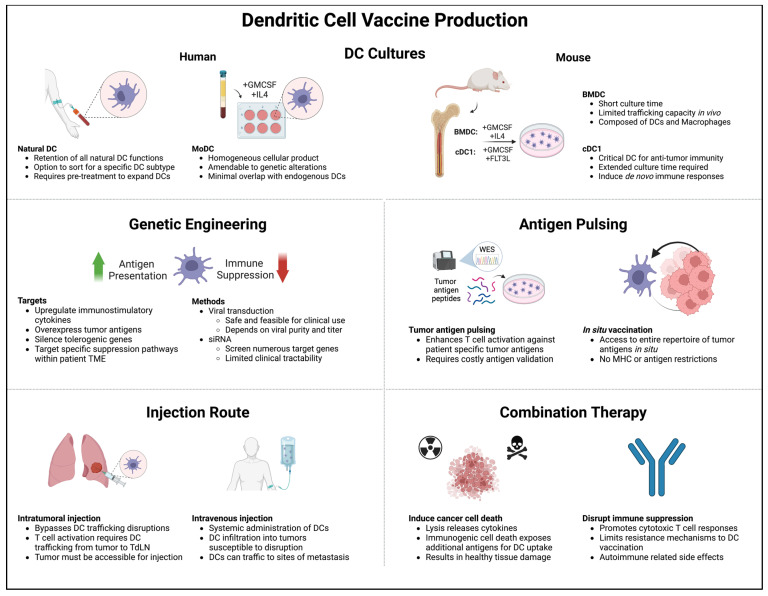
Major considerations when constructing a DC vaccine: (1) DC generation method, (2) genetic engineering, (3) antigen pulsing, (4) injection route, and (5) combination therapy. Tailoring these considerations to patient-specific TME phenotypes can overcome immunosuppression and improve vaccine-induced immune responses.

The majority of DC vaccines studied in early-phase clinical trials have not been able to obtain regulatory approval [174,180,198]. The clinical efficacy of most of these products has unfortunately been limited, though some standout cases of complete responses has fostered decades of continued research toward optimizing DC cancer vaccination. The optimal strategy in terms of DC production, modification, and administration route remain unclear (Figure 3), and the development of modern DC culture techniques and patient-specific antigen identification tools adds further complexity to vaccine design. However, multiple studies have been able to robustly demonstrate significant changes in the TME in response to DC vaccines, indicating that these treatments hold promise as potential tools in the immunotherapy armament (Table 2).

### 5.1. Changes in Cytokine Profiles

The rapid turnover of DCs limits the duration that injected DCs can survive in vivo [199]; thus, effective DC vaccination therapy cannot solely rely on the functions of injected DCs alone. To orchestrate a prolonged anti-tumor immune response, DC vaccination must alter cytokine secretion and soluble immune signals within the TME. In this section, we will summarize how DC tumor vaccination alters the endogenous cytokine profile within the TME toward an anti-tumor signature.

Numerous clinical trials treating patients with NSCLC with DC vaccine monotherapy have reported significant increases in IFNγ secretion from circulating patient-derived lymphocytes; however, increased secretion is seldom correlated with improved NSCLC survival [200,201,202,203,204]. In NSCLC and other cancers, increases in TNFɑ alone and TNFɑ, IFNγ, and IL2 together are associated with increased Th1 polarization post DC vaccination [156,205,206]. Sipuleucel-T DC vaccination has been shown to upregulate APC and activated-T cell cytokine secretion cytokine secretion in PBMCs [207]. The inclusion of the PAP prostate cancer antigen in the Sipuleucel-T DC cultures has been shown to significantly promote the secretion of key stimulatory cytokines such as IL12-p70, TNFɑ, IFNγ, and IL2. Productive DC antigen presentation is associated with the release of inflammatory cytokines such as IFNγ and TNFɑ, both of which are significantly upregulated in PBMCs co-cultured with mutant peptide-pulsed DCs compared to WT peptide-pulsed DCs [156,207]. Changes in the soluble immune factors associated with autologous DC vaccination and autologous tumor cell vaccination were compared in patients with melanoma [208]. The two patient cohorts showed similar levels of the 110 proteomic markers measured at baseline, but DC vaccination was found to be associated with innate, Th1, and Th17 responses, while autologous tumor vaccination was associated with only innate and Th2 responses. Despite these results demonstrating that DC-based vaccination results in pro-inflammatory cytokine secretion associated with Th1 responses and cytotoxicity, limited clinical responses indicate that the modification of DC-based vaccines could yield robust immune responses in treated populations.

A preclinical DC vaccine study treating LLC tumor-bearing mice with idoleamine 2,3-dioxygenase 2 (*Ido2*)-silenced DCs precisely quantified changes in serum cytokine concentrations induced by this modified DC vaccination [173]. *Ido2* was chosen as a gene-silencing target because its expression in DCs has been shown to induce tolerance via promoting the generation of CD4^+^ Treg cells [209]. Mice treated with the *Ido2*-silenced DC vaccine had significantly higher concentrations of IFNγ and TNFɑ with concurrent decreases in TGFβ and IL10 compared to untreated mice or mice treated with control DCs. The shifts in cytokine profiles were highly associated with an effective anti-tumor immune response. A preclinical proof of concept study that led to the CCL21-DC phase 1 clinical trial mentioned previously utilized DCs transduced with an adenovirus expressing CCL21 (DC-AdCCL21) to intratumorally vaccinate mice bearing NSCLC tumors [159]. The DC-AdCCL21 treatment led to significant increases in the intratumoral concentrations of GMCSF, IFNγ, CXCL9, CXCL10, and IL12 and significant decreases in immunosuppressive factors such as PGE2 and TGFβ. Clearly, modifications to DC vaccines can remodel the soluble immune mediators both systemically and within the TME. These data have led researchers to combine DC therapies with alternative cellular therapies to improve anti-tumor immune responses.

Various clinical trials have demonstrated the efficacy of DC + cytokine-induced killer cell (CIK) combination therapy for several forms of cancer [210]. CIK cultures are a heterogenous cell population derived from patient PBMCs and cultured in the presence of IFNγ, IL2, and anti-CD3 for two to three weeks prior to infusion into patients [211]. The primary subsets of cells within CIK cultures are CD3^+^ CD56^−^ T lymphocytes and CD3^+^ CD56^+^ NK T cells, the latter of which is thought to be the primary driver of cellular cytotoxicity within CIK cultures. In a DC + CIK clinical trial, patients with NSCLC were treated with either three or more or less than three cycles of combination therapy [212]. Among the patients treated with three or more cycles, the post-therapy serum concentrations of IL10 and TGFβ were significantly decreased compared to pre-treatment levels, which was not observed in patients treated with fewer than three cycles. Furthermore, increased cycle treatment resulted in a significant decrease in NSCLC recurrence compared to patients treated with fewer than three cycles.

**Table 2:** Selected DC vaccine clinical trials with immune monitoring data.

Vaccine Description	Clinical Study Design	Immune Monitoring	Ref.
ID MoDCs pulsed with tumor cell line lysate	Phase I in stage I-IIIB NSCLC after definitive therapy	Increased T cell IFNγ response to tumor lysate in 6/16 and 9/14 patients across two reports	[201,204]
LN injection of MoDCs pulsed with pleural effusion tumor lysate	Phase I in advanced refractory NSCLC	Increased T cell IFNγ response to tumor lysate in 3/8 patients	[200]
ID MoDCs pulsed with tumor lysate	Phase I in advanced refractory NSCLC	Increased T cell IFNγ response to tumor lysate in 5/9 patients	[202]
IV MoDCs and CIKs (<3 vs. ≥3 cycles)	Non-randomized study in resected NSCLC	Lower Treg frequency and IL10/TGFβ levels with ≥3 cycles	[212]
IV MoDCs pulsed with MUC1 and survivin	Phase I in resected NSCLC	Decreased Tregs; lower levels of TNFɑ and IL6 in 2/15 patients	[205]
IT MoDCs transduced with CCL21	Phase I in advanced refractory NSCLC	Increased T cell IFNγ response to TAAs in 6/16 patients; induced tumor T cell infiltration in 7/13	[185]
ID MoDCs pulsed with MAGE3 and survivin	Single-arm study in stage I-IIIB NSCLC after definitive therapy	Increased IFNγ production by peripheral T cells	[203]
IV/ID MoDCs transfected with TAAs	Phase I in GBM and NSCLC with brain metastases	Induced T cell responses to TAAs in 7/7 patients tested	[213]
ID MoDCs transduced with WT p53	Phase I/II in untreated SCLC as maintenance after chemotherapy	Improved T cell response to p53 in 18/43 patients; fewer responses in those with elevated MDSCs	[186]
ID MoDCs transduced with WT p53 +/− ATRA	Phase I in untreated SCLC as maintenance after chemotherapy	Increased T cell IFNγ response to p53 in 3/15 patients; 5/12 in ATRA combination arm	[214]
ID MoDCs pulsed with MAGE-1 peptide	Single-arm study in metastatic melanoma	Induced TIL cytolytic activity against autologous tumor cells in 2/2 patients	[151]
IV MoDCs pulsed with neoantigen peptides	Phase I in melanoma after progression on ICB	Developed new T cell responses to neoantigens in 3/3 patients and a more diverse TCR repertoire	[215]
SC MoDCs pulsed with tumor antigens vs. irradiated tumor cells	Randomized phase II in metastatic melanoma	DCs associated with increase in Th1/Th17 serum cytokines	[208]
ID MoDCs pulsed with melanoma cell lysates	Phase I-II in advanced colorectal cancer	Patients with SD had higher plasma levels of GM-CSF, TNFɑ, IFNγ, IL2, and IL5	[206]

MoDCs—monocyte-derived dendritic cells, ID—intradermal, LN—lymph node, IV—intravenous, IT—intratumoral, SC—subcutaneous, TAAs—tumor-associated antigens, NSCLC—non-small-cell lung cancer, TIL—tumor-infiltrating lymphocytes, CIKs—cytokine-induced killer cells, GBM—glioblastoma multiforme, SCLC—small-cell lung cancer, WT—wild-type, ATRA—all-trans retinoic acid, ICB—immune checkpoint blockade, TCR—T cell receptor, SD—stable disease.

### 5.2. Changes in Myeloid Populations

DC vaccines can drive the recruitment, reorganization, and polarization of endogenous myeloid subsets toward a phenotype more favorable for anti-tumor immune responses. In this section, we will review the intratumoral trafficking patterns of different DC subsets within vaccine injections and how DC vaccination affects endogenous myeloid cell populations.

Evidence supporting the effectiveness of cDC1 clinical vaccination is largely based on preclinical studies. One pre-clinical study investigated the differences in immune responses associated with CD103^+^ cDC1- and BMDC-based vaccines [167]. The CD103^+^ cDC1-based vaccine exhibited superior anti-tumor efficacy compared to the BMDC-based vaccine and was capable of protecting against melanoma metastases formation. Additionally, the cDC1 vaccine displayed superior trafficking to the TdLN; forty hours after vaccination, only the CD103^+^ cDC1s were found to be significantly increased in the TdLN, while BMDCs were undetectable. A separate study independently confirmed the superior migratory phenotype of cDC1s when utilized as a tumor vaccine. *Irf8* +32^−/−^ mice (which lack endogenous cDC1s) bearing 1956 mOVA tumors were intratumorally injected with a cDC1- or BMDC-based vaccine [168]. The TdLN was analyzed forty hours after vaccination, and while injected cDC1s were detected, BMDCs from the vaccine source were undetectable. Furthermore, only the cDC1-based vaccine was capable of driving anti-tumor immune responses in *Irf8* +32^−/−^ mice, demonstrating that cDC1-based vaccines can function independently of host cDC1s.

While improved retention, activation, and survival of injected DCs are phenotypes of an effective DC vaccine, changes in endogenous myeloid cells dictate the long-term outcome. Remodeling of the endogenous pool of myeloid cells both systemically and within the TME has been observed in several different DC vaccination studies. The FDA-approved Rose Bengal (RB) dye has been utilized as an eyedrop to identify damaged cells within the eye [216]. Recently, it has been shown that RB dye can induce the immunogenic cell death of cancer cells, which can lead to enhanced tumor antigen presentation and subsequent recognition [217]. A study evaluating RB dye and BMDCs as an intratumoral combination therapy found that this therapy was significantly efficacious in a murine LLC NSCLC model [218]. Flow cytometry phenotyping revealed that the combination therapy significantly decreased the number of M2 macrophages in the spleen and TME. Additionally, combination therapy significantly decreased MDSCs in the TME compared to control treatment. Other studies have shown that factors capable of inducing immunogenic cell death synergize with DC vaccination. The intratumoral injection of iPSC-derived DCs in combination with radiation therapy (RT) was evaluated for treating the poorly immunogenic AT3 murine breast cancer model [165]. In mice bearing fluorescent GFP^+^ AT3 tumors, combination treatment significantly increased the number of GFP^+^ DCs within the TdLN, suggesting that tumor cell death induced by RT enhances the capacity of iPSC-DCs to traffic tumor antigens. Furthermore, PD-L1 expression was significantly increased in intratumoral macrophages and DCs when RT was added to the DC vaccine.

A small-cell lung cancer (SCLC) clinical trial tested whether the inhibition of MDSC populations could improve immune responses associated with DC vaccination therapy [214]. All-trans retinoic acid (ATRA) has been shown to induce apoptosis in PMN-MDSCs and skew M-MDSC differentiation toward alternative myeloid cell populations [219], and ATRA has been used in combination with DC vaccination. For example, in one study, patients were evenly divided and treated with one of three arms: standard of care treatment or observation, DC vaccine only, or DC vaccine plus ATRA treatment. The combination therapy was well tolerated and displayed signs of enhanced efficacy. Only the combination therapy significantly decreased both Lin^−^ HLA-DR^−^ CD33^+^ and CD11b^+^ CD14^−^ CD33^+^ MDSC populations. Additionally, combination therapy significantly increased the number of patients with T cell responses against previously untargeted TAAs and significantly increased circulating granzyme B-positive CD8^+^ T cells. These results suggest that depleting MDSCs can improve DC vaccine immune responses. Further studies investigating this combination should be carried out, as it is possible that the most potent form of DC vaccination may require initial treatments to ablate suppressive myeloid cells prior to DC delivery.

### 5.3. Induction of Tumor-Specific T Cell Responses

The generation of T cell clones with TCR specificity against cancer neoantigens or TAAs is one of the primary goals of DC vaccination. Recent advances in next-generation sequencing and TCR detection tools have enabled researchers to demonstrate the induction of antigen-specific anti-tumor T cell responses post-DC vaccination. This expansion is the result of functional antigen presentation and serves as the best evidence of DC vaccine functionality. This section illustrates how DC vaccines induce patient-specific immune responses tailored to detect and kill cancer cells.

A 2021 NSCLC DC vaccination clinical trial leveraged modern in silico neoantigen prediction to pulse autologous MoDCs with putative immunogenic neoantigens derived from patient tumors [156]. Neoantigen-specific T cell responses were markedly improved after treatment, and an ELISPOT assay of patient PBMCs pulsed with mutant and matched WT peptides revealed significant T cell activation only in the presence of the predicted mutant peptides. CDR3 TCR-β chain sequencing performed on patient PBMCs co-cultured with either neoantigen or matched WT peptides revealed a significant decrease in TCR diversity and a significant increase in mean clone frequency in the PBMCs incubated with neoantigens. A significant increase in TCR convergence among the PBMCs incubated with neoantigens compared to WT incubation was also found, a surprising result that indicates that a neoantigen-pulsed DC vaccine can promote the expansion of T cells targeting identical antigens with unique TCR sequences. A similar personalized DC vaccine approach was used to treat three patients with stage III resected cutaneous melanoma [215]. This investigation pulsed MoDCs with seven predicted neoantigens before intravenous administration. All three patients displayed the robust expansion of CD8^+^ T cells specific for the neoantigens, as well as the expansion of T cells against naturally occurring neoantigens not pulsed in the vaccine product. These data suggest that this personalized DC vaccine approach can bolster anti-tumor immune responses through two potential mechanisms: the induction of antigen-specific CD8^+^ T cells targeting neoantigens pulsed in the DC vaccine and the generation of new antigen-specific CD8^+^ T cells with TCR specificity against previously untargeted cancer epitopes.

A 2020 DC vaccination clinical trial utilized DCs pulsed with TAAs to treat patients with either advanced lung cancer or glioblastoma [213]. Unlike the trials detailed above, the use of TAAs bypassed the often unreliable and laborious process of neoantigen prediction. TAA expression was confirmed from each patient’s tumor RNA. Three to seven different TAA mRNAs were electroporated into patient-derived MoDCs prior to infusion, and patients were treated with a combination of cyclophosphamide, poly I:C, imiquimod, and anti-PD-1 ICB. The patients with NSCLC who received treatment exhibited favorable clinical outcomes, including improved overall survival. Notably, one patient displayed the induction of antigen-specific CD4^+^ and CD8^+^ T cell responses against all the TAAs loaded into their DC vaccine. One unique form of DC vaccination that has been shown to induce antigen-specific T cell responses does not directly inject live DCs into patients. Rather, an antibody targeting a DC specific surface marker, such as C-type lectin domain containing 9A (Clec9a), is conjugated to a tumor antigen and injected into patients to promote the delivery of immunogenic tumor antigens to key DC subsets [220,221,222]. Although this approach is not an example of direct vaccination with live DC cells, its clinical feasibility and off-the-shelf applicability should be further explored in future studies.

Despite the tumor-specific T cell responses initiated by these vaccines, antigen-specific approaches can be resource-intensive and susceptible to resistance mechanisms. Targeted therapies foist enormous evolutionary pressure on cancer cells and have historically resulted in therapeutic resistance [223,224]. Antigen-agnostic in situ vaccination has the capacity to continuously present tumor antigens in vivo, giving this DC therapy platform the unique ability to engage multiple cancer immunity cycles and “keep up” with the evolving tumor clonality and immunogenicity to prevent immune escape. The aforementioned trial of a CCL21 gene-modified MoDC in situ vaccination was shown to induce TAA-specific T cell responses in a subset of patients with NSCLC, demonstrating the ability of DCs to promote these responses even in the absence of peptide pulsing [179].

Taken together, these data indicate that DC vaccines can induce the expansion of antigen-specific T cell responses to increase the breadth of cancer-specific T cell responses. Modern DC vaccination therapies combined with effective cancer neoantigen prediction have great potential to become a clinical standard for personalized cancer medicine.

## 6. Conclusions

The NSCLC TME is host to a wide variety of immunostimulatory and immunosuppressive cell populations capable of influencing tumor growth. DCs’ direct engagement with tumor cells via antigen uptake and presentation, along with their ability to interface with immune cells in the TME to promote anti-tumor immune signaling, makes them an ideal cellular vaccination candidate. Clinically, DC vaccination can induce the expansion of tumor-specific T cells, decrease immunosuppressive myeloid and lymphocyte populations within the TME, and alter the cytokine milieu towards a cytotoxic signaling signature. Although the clinical outcomes associated with DC vaccination treatment have shown great promise, this treatment modality has not yet revolutionized NSCLC standard of care. DC vaccine therapy strategies continue to develop as technologies that probe patient-specific tumor immunosuppression advance. Some of the most promising new data in the field of DC vaccination have come from studies in which the therapy was tailored to best engage patient-specific TME immune suppression. Trials in which DC vaccines are pulsed with tumor neoantigens or combined with drugs to neutralize immunosuppressive cells are the beginning stages of the next generation of DC vaccines. The future of NSCLC DC vaccine therapy is tightly intertwined with the future of cancer therapies targeting patient-specific TME features.

## Figures and Tables

**Table 1:** Selected DC vaccination preclinical studies utilizing different DC types and modifications.

Vaccine	Preclinical Efficacy	Mechanistic Findings	Ref.
IV BMDCs	Pulsing with TAA expressed by target tumor cell line improved efficacy	Splenocytes showed cytolytic activity against tumor cells after vaccine therapy	[152]
SC BMDCs	Pulsing with gp96 improved efficacy compared to non-pulsed DC or gp96 alone	Antitumor effect abrogated via the depletion of NK cells and CD8^+^/CD4^+^ T cells	[153]
SC BMDCs	Pulsing with both TAA- and MHC-II peptides proved more efficacious than using only one peptide pool	Induced stronger IFNγ response by CD8^+^ T to tumor antigens; Tregs decreased in spleen	[154]
ID BMDCs	Pulsing with MUC1-PD-L1 fusion protein improved antitumor efficacy	Induced splenic T cell activation and cytokine secretion; increased serum anti-PD-L1 antibody titers	[155]
IV BMDCs	Pulsing with neoantigen peptide improved efficacy compared to non-pulsed DCs	Increased tumor infiltration via IFNγ-producing CD8^+^ T cells	[156]
IV BMDCs	Transfection with E7 or p53 genes improved antitumor efficacy	Improved tumor-specific lysis and IFNγ production in splenocytes	[157]
IV BMDCs	Transfection with tumor total RNA proved more efficacious than pulsing with tumor lysate	Serum Th1 cytokines increased with therapeutic vaccination	[158]
IT BMDCs	Transduction with CCL21 improved antitumor efficacy	Efficacy diminished via IFNγ, CXCL9, or CXCL10 depletion; activity seen in contralateral tumors	[159]
ID BMDCs	Transduction with CCR7 promoted mature DC phenotype	CCR7-DCs showed greater migration to lymph nodes	[160]
SC BMDCs	Transduction with human livin α improved efficacy	Induced cytolytic activity against tumor cells in splenic T cells	[161]
IT BMDCs	Transduction with GITRL and pulsing with tumor cell lysates proved more efficacious than pulsing alone	Increased IFNγ-producing CD8^+^ T cells and deceased Tregs in the spleen	[162]
SC BMDCs	Transduction with CK19 improved antitumor efficacy	Spurred T cell proliferation in vitro; induced cytolytic activity against tumor cells in splenic T cells	[163]
IT and IV BMDCs	Transduction with OVA improved response against OVA-expressing tumors	T cell proliferation and cytolytic activity improved in DC co-culture	[164]
IT iPSC-DCs and RT	iPSC-DC vaccine was synergistic with RT in treating tumors	iPSC-DCs resembled cDC2s; RT induced DC trafficking to TdLN and increased DC/CD8^+^ T cell aggregates	[165]
ID cDC1s	cDC1 vaccine pulsed with tumor cell lysate was synergistic with anti-PD-1 in treating tumors	Enhanced activation of TdLN T cells; increased tumor T cell infiltration	[166]
IT cDC1s	cDC1 vaccine pulsed with OVA or tumor lysate proved more efficacious than BMDC vaccine	Increased tumor and TdLN infiltration by antigen-specific and IFNγ-producing T cells	[167]
IT cDC1s	cDC1 vaccine proved more efficacious than BMDCs in a cDC1-deficient model	cDC1s migrated to TdLN; increased splenic antigen-specific T cells; efficacy seen in contralateral tumors	[168]
SC pDCs and mDCs	A mix of pDCs and mDCs pulsed with a OVA peptide proved more efficacious than either vaccine alone	pDCs increased peripheral antigen-specific T cells; mixed vaccine requires mDC but not pDC MHC-I expression	[169]

BMDCs—bone marrow-derived dendritic cells, IV—intravenous, SC—subcutaneous, IT—intratumoral, ID—intradermal, TAAs—tumor-associated antigens, MHC—major histocompatibility complex, OVA—ovalbumin, GITRL—glucocorticoid-induced tumor necrosis factor receptor, iPSC-DCs—induced pluripotent stem cell-derived dendritic cells, RT—radiation therapy, cDC—conventional dendritic cells, TdLN—tumor-draining lymph node, pDC—plasmacytoid dendritic cells, mDC—myeloid dendritic cells.

## Data Availability

No new data were created or analyzed in this study. Data sharing is not applicable to this article.
